# Elevated Leukocyte Glucose Index (LGI) Is Associated with Diabetic Ketoacidosis (DKA) Severity and Presence of Microvascular Complications

**DOI:** 10.3390/medicina61050898

**Published:** 2025-05-15

**Authors:** Mircea Cătălin Coșarcă, Raluca Maria Tilinca, Nicolae Alexandru Lazăr, Suzana Vasilica Șincaru, Bogdan Corneliu Bandici, Cosmin Carașca, Ráduly Gergő, Adrian Vasile Mureșan, Mariana Cornelia Tilinca

**Affiliations:** 1 Department of Anatomy, George Emil Palade University of Medicine, Pharmacy, Science and Technology of Targu Mures, 540139 Targu Mures, Romania; catalin.cosarca@umfst.ro (M.C.C.);; 2 Clinic of Vascular Surgery, Mures County Emergency Hospital, 540136 Targu Mures, Romania; 3 Clinic of Epidemiology, Mures County Emergency Hospital, 540136 Targu Mures, Romania; raluca.tilinca@gmail.com; 4 Department of Forensic Medicine, George Emil Palade University of Medicine, Pharmacy, Science and Technology of Targu Mures, 540139 Targu Mures, Romania; cosmin.carasca@umfst.ro; 5 Department of Vascular Surgery, George Emil Palade University of Medicine, Pharmacy, Science and Technology of Targu Mures, 540139 Targu Mures, Romania; 6 Discipline of Internal Medicine, Department ME2, Faculty of Medicine, “George Emil Palade” University of Medicine, Pharmacy, Science and Technology, 540142 Targu Mures, Romania; mariana.tilinca@umfst.ro

**Keywords:** diabetic ketoacidosis, DKA, leukocyte glucose index, LGI, biomarkers

## Abstract

*Background and Objectives*: Diabetic ketoacidosis (DKA) represents the most prevalent hyperglycemic emergency and poses a significant life-threatening metabolic risk for individuals with diabetes. The present study examines the predictive role of the leukocyte glucose index (LGI) values at baseline in diagnosing the severity of DKA and their correlation with the presence of diabetes-related microvascular complications. *Materials and Methods*: A retrospective observational study was conducted involving a total of 94 patients who had previously confirmed diagnoses of either Type I or Type II diabetes mellitus and presented with ketoacidosis upon emergency admission to the Department of Diabetology, Nutrition, and Metabolic Disease. Demographic information, values of arterial systolic and diastolic pressure, known duration and type of diabetes, severity of ketoacidosis, routine laboratory results, and blood gas analyses were retrieved from the hospital’s electronic database. *Results*: Higher diastolic blood pressure (DBP) values were observed in both mild (*p* = 0.021) and severe DKA (*p* = 0.035) compared to moderate DKA. When examining laboratory data, elevated white blood cell (WBC) counts were observed in severe DKA when compared to mild DKA (*p* = 0.009), as well as increased neutrophil counts in both moderate (*p* = 0.038) and severe (*p* = 0.011) DKA relative to mild DKA. Furthermore, patients with severe DKA exhibited lower values of venous blood pH, partial pressure of carbon dioxide (pvCO_2_), base excess (BE), and bicarbonate than the other groups (all *p* < 0.05), alongside higher levels of lactate, anion gap, and LGI (all *p* < 0.05). Regarding the parameters of arterial blood gas, we identified a negative correlation between LGI values and venous blood pH (r = −0.383, *p* < 0.001), serum bicarbonate (r = −0.352, *p* < 0.001), pCO_2_ (r = −0.271, *p* = 0.009), and BE (r = −0.330, *p* < 0.001). At univariate analysis, elevated LGI values are associated with the severity of DKA (OR: 1.87, *p* = 0.016) and diabetes-related microvascular complications (OR: 2.16, *p* = 0.010). *Conclusions*: The positive correlation between LGI and DKA severity and between LGI and diabetes microvascular complications highlights the potential utility of LGI as a predictive marker, facilitating early risk stratification and clinical decision-making.

## 1. Introduction

Diabetic ketoacidosis (DKA) represents the most prevalent hyperglycemic emergency and poses a significant life-threatening metabolic risk for individuals with diabetes [1]. This condition primarily results from an insulin deficiency and elevated levels of counter-regulatory hormones such as glucagon, cortisol, and catecholamines. The classical triad of DKA symptoms includes hyperglycemia (blood glucose > 250 mg/dL), ketosis (≥3 mmol/L), and anion gap metabolic acidosis (pH < 7.3 or bicarbonate [HCO_3_] < 15–18 mmol/L). Moreover, patients experiencing DKA may exhibit distinctive signs and symptoms like fruity-smelling breath (due to increased ketone levels), tachycardia, Kussmaul respirations, and altered mental status [2,3,4].

DKA is primarily seen in those with type I diabetes, but it can also affect individuals with type II diabetes, particularly under specific circumstances like infections, significant trauma, surgery, or the use of certain medications [1,2,5]. Furthermore, according to publications, while individuals diagnosed with type I diabetes possess a greater risk of developing diabetic ketoacidosis (DKA), both types of diabetes exhibit an elevated incidence of DKA risk [6,7,8].

The American Diabetes Association (ADA) categorizes DKA into mild, moderate, and severe forms, based on blood glucose levels, ketonemia, acidosis severity, mental status, and the required level of care. In mild DKA, blood glucose is at least 200 mg/dL (11.1 mmol/L), β-hydroxybutyrate levels range from 3.0 to 6.0 mmol/L, and acidosis is characterized by a pH between 7.25 and 7.30 or bicarbonate levels between 15 and 18 mmol/L. Patients remain alert and can typically be managed in a regular or observation nursing unit. Moderate DKA also presents with blood glucose of at least 200 mg/dL and β-hydroxybutyrate levels between 3.0 and 6.0 mmol/L, but acidosis is more pronounced, with a pH between 7.0 and 7.25 and bicarbonate levels ranging from 10 to less than 15 mmol/L. Patients in this category may be alert or drowsy, requiring care in a step-down unit or intermediate care setting. Severe DKA, the most critical form, involves blood glucose levels of at least 200 mg/dL, β-hydroxybutyrate levels exceeding 6.0 mmol/L, and severe acidosis with a pH below 7.0 and bicarbonate levels lower than 10 mmol/L. Mental status may deteriorate into stupor or coma, necessitating admission to an intensive care unit (ICU). It is important to note that not all criteria must be met to classify a patient’s DKA severity, and the final decision on classification and level of care is usually based on clinical judgment [3,5].

In Romania, the prevalence of diabetes is about 11.6%, according to the PREDATORR study [4], and this number is steadily increasing, with many cases remaining undiagnosed. Given these factors, diabetes and its complications pose ongoing challenges for diagnosis, treatment, and long-term management [3,4,9]. The latest guidelines suggest a proactive strategy for every newly diagnosed patient with diabetes mellitus, particularly to prevent severe complications like DKA [3,9,10].

In addition to metabolic dysfunctions, DKA triggers a systemic inflammatory response characterized by increased concentrations of various cytokines, including interleukin-1 beta (IL-1B), interleukin-6 (IL-6), interleukin-8 (IL-8), interleukin-10 (IL-10), and tumor necrosis factor-alpha (TNF-α) [11,12,13,14,15,16,17,18,19,20,21,22]. This inflammatory cascade results in cellular activation, heightened levels of oxidative stress, and endothelial damage, potentially exacerbating patient outcomes, particularly in those who are not treated appropriately and in a timely manner [10,14,15]. Numerous studies suggested that leukocytes, notably neutrophils and monocytes, exhibit significant sensitivity to fluctuations in glucose levels [12,13]. A condition of hyperglycemia amplifies the inflammatory response, thereby activating leukocytes and facilitating the release of pro-inflammatory cytokines [3,10,15]. Recently, a novel biomarker has been proposed and analyzed: the leukocyte glucose index (LGI), which is derived from the integration of peripheral blood glucose concentrations and total leukocyte counts [17,18,19,20,21,23,24,25,26,27,28]. This index reflects the intricate interplay between glycemic regulation and the host’s immune and inflammatory responses. This dualistic composition renders LGI a potentially robust inflammatory biomarker, exhibiting a significant correlation with adverse clinical sequelae in patients presenting with DKA [17,18,19,20,21,23,24,25,26,27,28].

The LGI effectively encapsulates both acute hyperglycemia and leukocytosis-mediated immune activation, thus facilitating the early stratification of patients at elevated risk for severe complications. Furthermore, the prognostic utility of the LGI transcends the confines of diabetes-related conditions. This is also pictured in the papers published recently around cardiovascular disease and LGI use in the prognostic of things like the severity of coronary artery disease [20], the prognostic of postoperative period of coronary artery bypass grafting [21], and the severity of COVID-19 infection [22].

According to the current literature, the relationship between diabetes type and the severity of DKA remains ambiguous [29,30,31,32,33]. While some studies found no significant differences in DKA severity between type I and type II diabetes patients [29,31], other studies indicate that type II diabetes mellitus patients may have a heightened risk for severe DKA [30]. Conversely, additional research suggests that individuals with type I diabetes are also at increased risk for severe DKA [32]. Therefore, it is essential to propose and validate new tools for assessing DKA severity and diabetes-related microvascular complications, which will enable better patient stratification and facilitate the development of optimal management strategies.

The present study seeks to examine the predictive significance of LGI values in the diagnosis of the severity of DKA and their correlation with the presence of diabetes-related microvascular complications. In addition, this study aims to explore the risk factors pertinent to the aforementioned outcomes.

## 2. Materials and Methods

### 2.1. Study Design

A retrospective observational study included all patients with a confirmed diagnosis of either Type I or Type II diabetes mellitus, all of whom presented with ketoacidosis during emergency admissions to the Department of Diabetology, Nutrition, and Metabolic Disease at Targu Mures County Emergency Hospital from January 2020 to December 2023. We excluded patients under 18 years old, those presenting with septic shock, and individuals with known autoimmune or systemic inflammatory diseases. Furthermore, we excluded patients with confirmed COVID-19 infection at admission, as the pandemic adversely affected patient outcomes [34,35,36,37,38,39] and systemic inflammatory status [40,41,42,43,44,45].

### 2.2. Data Collection

Once all eligible patients were identified and selected, demographic information, values of the arterial systolic and diastolic pressure, known duration and type of diabetes, severity of ketoacidosis, as well as routine laboratory results and blood gas analyses were retrieved from the hospital’s electronic database. Laboratory analyses were performed at the Central Laboratory of the Emergency Clinical County Hospital in Târgu Mureș, while blood gas analysis was conducted in the Emergency Department. Height and weight were measured at admission and BMI was calculated for every patient using the following formula: BMI = weight (in kg)/height^2^ (in m^2^).

Patients were classified into 3 different groups, according to the severity of DKA—mild, moderate, and severe, in accordance with the ADA classification. Furthermore, the microvascular complications associated with diabetes were characterized by the presence of a minimum of two out of the three complications observed: nephropathy, retinopathy, or neuropathy at the time of hospitalization. For systemic inflammatory markers, we calculated and used the leuko-glycemic index, according to the formula: the leukocyte count (in 10^3^/uL) multiplied by the glucose level (in mg/dL), and the result divided by 1000.

### 2.3. Study Outcome

The primary objective of the current study was to determine if admission LGI, an inflammatory biomarker, could predict the evolution and severity of DKA and could help generate a different approach and individualized treatment for each patient.

### 2.4. Statistical Analysis

Statistical analysis was conducted using SPSS for Mac OS version 28.0.1.0, developed by SPSS, Inc. in Chicago, IL, USA. Continuous variables were expressed as means with standard deviations (SD), while categorical data variables were presented as frequencies and percentages. Chi-square tests were used to compare dichotomous variables between groups, while Mann–Whitney and Student’s *t*-tests were applied to assess differences in continuous variables. ROC curve analysis was performed to determine the optimal cut-off values for LGI using the Youden index, which ranges from 0 to 1 and is calculated as Youden Index = Sensitivity + Specificity − 1. All tests were two-tailed, and a *p*-value of less than 0.05 was considered statistically significant.

## 3. Results

In the present study, a cohort of 94 patients was enrolled, with a mean age of 47.75 ± 18.02 years and a mean BMI of 39.9, of which 67.02% were male. Additionally, 48 patients (51.06%) were identified as having type I diabetes, while 46 patients (48.94%) were diagnosed with type II diabetes. Concerning the duration of diabetes, we recorded a mean duration of 6 years, with a minimum of 3 years and a maximum of 15 years. Upon admission, we documented a mean heart rate of 104 beats per minute (bpm), a systolic blood pressure (SBP) of 128 mmHg, and a diastolic blood pressure (DBP) of 78 mmHg. Furthermore, 7 patients (7.44%) were admitted with nephropathy, 16 patients (17.02%) exhibited retinopathy, and 35 patients (37.23%) showed signs of neuropathy upon admission. Furthermore, 15.95% of patients exhibited mild DKA, 57.45% displayed moderate DKA, and 26.60% presented with severe DKA (refer to Table 1). Additional information, along with laboratory data, is provided in Table 1.

Regarding demographic data, no differences were observed among the patient groups based on the severity of DKA. Additionally, there were no notable differences in BMI, duration of diabetes, pulse, and SBP (Table 2). However, higher DBP values were found in mild (*p* = 0.021) and severe DKA (*p* = 0.035) compared to moderate DKA. When examining laboratory data, elevated WBC counts were observed in severe DKA when compared to mild DKA (*p* = 0.009), as well as increased neutrophil counts in both moderate (*p* = 0.038) and severe (*p* = 0.011) DKA relative to mild DKA. Furthermore, patients with severe DKA exhibited lower values of venous blood pH, pvCO_2_, base excess, and bicarbonate than the other groups (all *p* < 0.05), alongside higher levels of lactate, anion gap, and LGI (all *p* < 0.05) (Table 2). Interestingly, the average LGI value is about 2.5 times greater in instances of severe diabetic ketoacidosis (12.52 vs. 5.64, *p* = 0.001) and nearly 2 times greater in cases of moderate diabetic ketoacidosis (9.41 vs. 5.64, *p* = 0.044) compared to mild diabetic ketoacidosis. It is noteworthy that there are no statistically significant differences among the three patient groups concerning glucose and WBC levels, with the exception of the observed differences between severe diabetic ketoacidosis and mild diabetic ketoacidosis.

**Figure 1:**
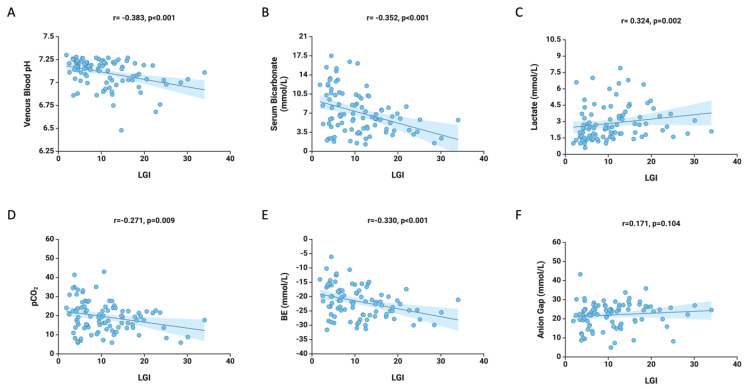
Correlations between LGI values at admission and venous blood pH (**A**), serum bicarbonate (**B**), lactate (**C**), pCO_2_ (**D**), BE (**E**), and anion gap (**F**) values.

Concerning the parameters of arterial blood gas, a negative correlation was observed between LGI values and venous blood pH (r = −0.383, *p* < 0.001), serum bicarbonate (r = −0.352, *p* < 0.001), pCO_2_ (r = −0.271, *p* = 0.009), and BE (r = −0.330, *p* < 0.001). Conversely, a positive correlation was established with lactate values (r = 0.324, *p* = 0.002) (Figure 1). No statistically significant correlation was observed between LGI values and the anion gap (r = 0.171, *p* = 0.104).

Additionally, an ROC curve analysis was conducted to evaluate the relationship between admission LGI values and study outcomes. The findings revealed a significant correlation between high LGI values and clinical outcomes, identifying a cut-off value of 10.43 (63.7% sensitivity and 56.1% specificity) for the severity of DKA (*p* = 0.002). Furthermore, a cut-off value of 11.07 (69% sensitivity and 64.3% specificity) was identified for the presence of diabetes-related microvascular complications (*p* = 0.013) (Table 3).

A univariate analysis was conducted to examine the association between LGI values and the outcomes documented in the present study. Thus, elevated LGI values are associated with the severity of DKA (OR: 1.87, *p* = 0.016) and diabetes-related microvascular complications (OR: 2.16, *p* = 0.010) (Table 4). In addition, increased WBC counts (OR: 1.75, *p* = 0.017), lactate levels (OR: 1.86, *p* = 0.009), and anion gap measurements (OR: 1.69, *p* = 0.047) are also associated with the severity of DKA. Moreover, higher venous blood pH (OR: 0.07, *p* < 0.001), base excess (OR: 0.03, *p* < 0.001), and bicarbonate concentrations (OR: 0.17, *p* < 0.001) serve as protective factors against DKA severity. Nevertheless, none of the aforementioned parameters were significantly associated with diabetes microvascular complications, as outlined in Table 4.

## 4. Discussion

To the best of our knowledge, the current study findings establish for the first time a significant correlation between elevated LGI values upon hospital admission and both the clinical severity of DKA and the co-existence of diabetic microvascular complications. DKA remains one of the most serious acute complications of diabetes mellitus, often presenting as a metabolic emergency requiring immediate intervention [1,2,46]. The complex interplay of hyperglycemia, ketosis, metabolic acidosis, and systemic inflammation constitutes the core pathophysiology of DKA, necessitating reliable prognostic markers for severity assessment and therapeutic guidance. LGI, an emerging biomarker, has demonstrated potential in evaluating DKA severity. This study aimed to investigate the correlation between LGI and DKA severity, exploring its potential clinical utility. We analyzed data from 94 patients with ketoacidosis, and the results of this study revealed several key findings.

The synergistic effect of elevated leukocyte count and hyperglycemia amplifies the inflammatory cascade, potentially exacerbating endothelial dysfunction and systemic oxidative stress. This observation aligns with previous research, highlighting the responsiveness of leukocytes, particularly neutrophils and monocytes, to glucose fluctuations, which triggers the release of pro-inflammatory cytokines such as IL-6, IL-8, and TNF-α [2,9,47]. The present study supports the hypothesis that LGI serves as a combined marker of metabolic stress and inflammatory burden, thereby facilitating risk stratification in DKA.

The role of inflammation in the pathogenesis of metabolic disorders is further supported by Raya-Cano et al., which highlights that Metabolic Syndrome (MetS) is characterized by chronic low-grade inflammation. The study indicates that white blood cell count (WBC) can be used as a marker of this inflammation, with higher leukocyte counts observed in individuals with MetS. This is consistent with the current study findings in DKA, where elevated LGI, reflecting increased leukocyte activity, correlates with disease severity [48].

Beyond its utility as a marker of glycemic control, LGI effectively mirrors the inflammatory state characteristic of DKA. The significant increase in neutrophil counts in patients with severe DKA, compared to those with milder forms, underscores the marked immune activation associated with hyperglycemic crises.

Moreover, this study data reveal a strong correlation between LGI and acid-base imbalances, including increased anion gap, decreased bicarbonate levels, and worsening metabolic acidosis. This suggests that heightened leukocyte activation may exacerbate acid-base disturbances, potentially contributing to cellular hypoxia and a poorer prognosis. This is consistent with the broader understanding of inflammation’s role in exacerbating metabolic derangements across different disease states [48,49].

Notably, the association between LGI and lactate levels in the current study suggests a link between inflammatory activation and tissue hypoxia in severe DKA. Lactic acidosis, a consequence of anaerobic metabolism due to circulatory compromise, is a well-established predictor of adverse outcomes in critically ill patients [50]. The observed correlation supports the notion that heightened inflammation, as indicated by LGI, may contribute to impaired perfusion and metabolic decompensation in severe DKA.

The significant association between LGI and microvascular complications points to the potential utility of this marker beyond acute DKA episodes. Chronic low-grade inflammation is a recognized driver of diabetic complications, including nephropathy, neuropathy, and retinopathy [48,51]. Given that LGI reflects systemic inflammatory activation, its potential as an early marker for identifying patients at risk of long-term complications calls for further investigation. The observed correlation with microvascular complications underscores the need for prospective studies evaluating the predictive value of LGI in chronic diabetes management.

Incorporating LGI into routine clinical assessments could enhance risk stratification in DKA patients, enabling tailored interventions. Patients with high LGI values may benefit from more aggressive fluid resuscitation, closer monitoring of inflammatory markers, and early adjunctive therapies targeting immune modulation. Additionally, further research should explore the impact of anti-inflammatory interventions in DKA management, particularly in patients with elevated LGI values, to determine whether targeting systemic inflammation improves metabolic and clinical outcomes.

This study, while trying to elucidate the potential of LGI as a predictive marker in DKA severity, is not without limitations that warrant consideration. The single-center design may constrain the generalizability of the findings to broader populations, and a larger, multicenter study would enhance the robustness of the conclusions. Another limitation of the current study is the lack of information related to underlying infections or other inflammatory conditions that may have increased the WBC and thus influenced the LGI values. A significant limitation of the present study is the insufficient information regarding the chronic antidiabetic treatments administered to the patients, as well as the patients’ adherence to these medications. Furthermore, the retrospective nature of the study precludes the establishment of causal relationships between LGI and DKA severity; future interventional or prospective studies are needed to determine whether modulating LGI can directly impact disease progression or outcomes. Future research should also focus on the longitudinal assessment of LGI in DKA patients, investigating its utility in predicting long-term complications.

## 5. Conclusions

This study underlines the inflammatory component of DKA, where increased leukocyte counts parallel worsening metabolic disturbances. This observation aligns with previous research emphasizing the role of systemic inflammation in DKA progression. The positive correlation between LGI and DKA severity, as well as between LGI and diabetes microvascular complications, highlights the potential utility of LGI as a predictive marker, facilitating early risk stratification and clinical decision-making. Moreover, the findings suggest that LGI may serve as a supplementary tool to conventional biochemical parameters, aiding in more accurate severity assessment and targeted management strategies. However, further research is necessary to establish standardized LGI thresholds and evaluate their prognostic significance in diverse patient populations.

## Figures and Tables

**Table 1:** Characteristics of patients enrolled in the current study.

Variables		All Patients n = 94
Age, mean ± SD		47.75 ± 18.02
Male, no. (%)		63 (67.02%)
Type I Diabetes, no. (%)		48 (51.06%)
Type II Diabetes, no. (%)		46 (48.94%)
Height (cm), median (Q1–Q3)		168 (165–175)
Weight (kg), median (Q1–Q3)		62 (57–70)
BMI, median (Q1–Q3)		21.48 (21.32–25.25)
Duration of Diabetes (year), median (Q1–Q3)		6 (3–15)
Heart Rate, median (Q1–Q3)		104 (92–120)
Systolic Blood Pressure, median (Q1–Q3)		128 (115–145)
Diastolic Blood Pressure, median (Q1–Q3)		78 (70–86)
Severity of DKA	Mild, no. (%)	15 (15.95%)
	Moderate, no. (%)	54 (57.45%)
	Severe, no. (%)	25 (26.60%)
Diabetes-Related Microvascular Complications	Nephropathy, no. (%)	7 (7.44%)
	Retinopathy, no. (%)	16 (17.02%)
	Neuropathy, no. (%)	35 (37.23%)
Laboratory data, median (Q1–Q3)		
WBC		16.04 (12.24–23.57)
Potassium, mmol/L		4.67 (4.13–5.6)
Sodium, mmol/L		134 (131–137)
Clor, mmol/L		104 (101–110)
Calcium, mmol/L		1.26 (1.16–1.32)
HbA1c, (%)		11.2 (10.02–12.47)
Glucose, (mg/dL)		530 (423–730)
BUN, (mg/dL)		59.91 (36.15–83.29)
Creatinine, (mg/dL)		1.71 (1.38–2.2)
Erytrocytes, ×10^6^/uL		4.7 (4.33–5.17)
Hemoglobin, g/dL		14.2 (13.12–15.87)
Hematocrit, %		42.35 (38.9–47.32)
Neutrophils, ×10^3^/uL		13.94 (9.78–20.34)
Lymphocytes, ×10^3^/uL		1.42 (1.01–2.38)
Serum albumin, g/dL		3.54 (3.11–3.81)
Total protein, g/dL		5.84 (5.33–6.21)
Total cholesterol, mg/dL		173.15 (138.82–204.15)
Triglycerides, mg/dL		130.1 (96.35–258.4)
Amylase, U/L		48 (34–86)
Total bilirubin, mg/dL		0.46 (0.28–0.66)
ALT, U/L		20.8 (13–27)
AST, U/L		22 (16–30)
Venous blood pH		7.15 (7.02–7.22)
PvCO_2_		19.45 (13.92–24.07)
PvO_2_		55.5 (40.85–102)
Base excess, mmol/L		−21.8 (−26.18–−17.47)
Lactate, mmol/L		2.4 (1.6–3.6)
Bicarbonate, mmol/L		6.35 (4.12–10)
Anion gap, mEq/L		23.4 (18.5–26.4)
LGI		9.8 (5.28–14.48)

**Table 2:** Demographic data, comorbidities, clinical data, and laboratory data according to the severity of DKA.

Variables	Mild n = 15	Moderate n = 54	Severe n = 25	*p* Value		
				1–2	1–3	2–3
Age mean ± SD	41.13 ± 19.01	50.05 ± 18.53	46.76 ± 15.77	0.188	0.459	0.556
Male no. (%)	8 (53.33%)	39 (72.22%)	16 (64.00%)	0.171	0.506	0.461
Type I Diabetes, no. (%)	9 (60.0%)	39 (72.22%)	16 (64.0%)	0.365	0.800	0.461
Type II Diabetes, no. (%)	6 (40.0%)	15 (27.78%)	9 (36.0%)			
Height (cm), median (Q1–Q3)	169 (163.5–173.75)	168 (165–173.25)	169 (163.5–175)	0.960	0.863	0.771
Weight (kg), median (Q1–Q3)	65 (60–69)	63.5 (57.75–70)	60 (55–65.5)	0.652	0.181	0.212
BMI, median (Q1–Q3)	22.98 (21.32–26.25)	23.09 (20.42–25.44)	21.24 (19.49–24.51)	0.821	0.313	0.279
Duration of Diabetes (year), median (Q1–Q3)	6 (2.5–16.5)	6.5 (2.25–18.75)	6 (3–13.75)	0.881	0.883	0.712
Pulse, median (Q1–Q3)	101 (94.5–120)	107 (87–118)	101 (92–115)	0.841	0.967	0.852
SBP, median (Q1–Q3)	130 (119.5–152.5)	127 (112–140)	127 (115–151)	0.265	0.676	0.435
DBP, median (Q1–Q3)	82 (79–86.5)	73 (70–80)	80 (72–94)	0.021	0.613	0.035
Nephropathy, no. (%)	3 (20.0%)	2 (3.70%)	2 (8.0%)	0.053	0.281	0.428
Retinopathy, no. (%)	2 (13.33%)	9 (16.67%)	5 (20.0%)	0.755	0.593	0.718
Neuropathy, no. (%)	5 (33.33%)	21 (38.89%)	9 (36.0%)	0.694	0.864	0.805
Laboratory data, median (Q1–Q3)						
WBC	12.61 (10.66–16.04)	15.35 (12.31–23.45)	20.39 (13.9–31.63)	0.090	0.009	0.134
Potassium, mmol/L	4.56 (4.42–5.09)	4.89 (4.09–5.6)	4.77 (4.14–5.6)	0.595	0.571	0.903
Sodium, mmol/L	133 (130–136)	134 (131–136.75)	135 (133–138)	0.542	0.100	0.138
Chlorine, mmol/L	104.3 (100.7–108.8)	102.9 (101–110)	106 (101–112)	0.875	0.660	0.435
Calcium, mmol/L	1.21 (1.12–1.27)	1.26 (1.17–1.32)	1.26 (1.19–1.34)	0.051	0.058	0.829
HbA1c, (%)	11.2 (10.02–12.47)	11.2 (10.02–12.47)	11.2 (10.02–12.47)	0.898	0.306	0.270
Glucose, (mg/dL)	504 (407–764)	515.5 (409–708)	595.5 (500–739.75)	0.889	0.223	0.142
BUN, (mg/dL)	40.66 (25.55–72.03)	62.25 (40.4–85.21)	60.8 (36.6–73.2)	0.082	0.304	0.476
Creatinine, (mg/dL)	1.36 (0.95–2.26)	1.7 (1.43–2.16)	1.89 (1.52–2.22)	0.319	0.189	0.567
Erythrocytes ×10^6^/uL	4.53 (4.41–4.85)	4.84 (4.25–5.21)	4.65 (4.34–5.36)	0.589	0.643	0.979
Hemoglobin, g/dL	13.8 (13.36–14.35)	14.3 (13.25–16.02)	14.8 (12.5–15.8)	0.314	0.474	0.805
Hematocrit, %	41.67 (39.85–42.95)	42.35 (38.9–46.5)	42 (38.3–49.21)	0.434	0.378	0.807
Neutrophils, ×10^3^/uL	9.78 (6.60–14.46)	13.94 (10.22–21.25)	16.5 (11.5–26.95)	0.038	0.011	0.348
Lymphocytes, ×10^3^/uL	1.49 (1.09–2.62)	1.28 (0.92–1.81)	1.9 (1.12–2.32)	0.280	0.874	0.280
Serum albumin, g/dL	3.66 (3.43–3.97)	3.54 (3.16–3.92)	3.23 (3.05–3.74)	0.387	0.150	0.367
Total protein, g/dL	5.92 (5.54–6.38)	6.05 (5.36–6.22)	5.53 (5.25–5.88)	0.696	0.195	0.166
Total cholesterol, mg/dL	188.1 (166.85–229.17)	155.1 (131.5–198.1)	170.4 (142.6–219.4)	0.102	0.314	0.512
Triglycerides, mg/dL	193 (105.35–352.05)	132.2 (107.4–258.4)	114 (80.6–165.8)	0.702	0.204	0.196
Amylase, U/L	47.75 (38.75–59)	44 (30–84)	68 (41–133.75)	0.907	0.144	0.063
Total bilirubin, mg/dL	0.28 (0.25–0.72)	0.52 (0.31–0.72)	0.38 (0.23–0.48)	0.076	0.586	0.005
ALT, U/L	18 (11.45–23.5)	19 (13–26.75)	24 (17–40)	0.540	0.036	0.037
AST, U/L	16 (9.65–28.5)	23 (17.1–29.5)	20.1 (15.9–42)	0.045	0.077	0.969
Venous blood pH	7.25 (7.19–7.26)	7.17 (7.11–7.22)	6.93 (6.87–6.99)	0.023	<0.001	<0.001
PvCO_2_	27.7 (22.25–34.8)	18.3 (13.85–22.77)	17.6 (12.2–21.4)	<0.001	<0.001	0.410
PvO_2_	35.4 (29.6–57.45)	59.5 (43.62–107.77)	57 (41–87.4)	0.008	0.066	0.478
Base excess, mmol/L	−14 (−18.2–−11.3)	−21.25 (−23.8–−17.75)	−27.8 (−29–−25)	0.003	<0.001	<0.001
Lactate, mmol/L	1.7 (1.6–2.45)	2.3 (1.6–3.4)	3.5 (2.5–4.6)	0.226	0.004	0.016
Bicarbonate, mmol/L	12.8 (9.15–15.3)	6.6 (5.03–9.65)	3.9 (3.1–5.9)	<0.001	<0.001	<0.001
Anion gap, mEq/L	18.8 (12.6–22.05)	23.79 (19.3–26.6)	25 (21.5–27.2)	0.009	<0.001	0.199
LGI	5.64 (3.93–9.69)	9.41 (5.28–13.19)	12.52 (10.43–17.24)	0.057	0.001	0.044

**Table 3:** ROC curve analysis of admission LGI value regarding DKA severity and diabetes microvascular complications.

Variables	Cut-Off	AUC	Std. Error	95% CI	Sensitivity	Specificity	*p* Value
**DKA Severity**							
LGI	10.43	0.688	0.066	0.565–0.816	63.8%	56.1%	0.002
**Diabetes Microvascular Complications**							
LGI	11.07	0.700	0.080	0.543–0.858	69%	64.3%	0.013

**Table 4:** The association of laboratory data and LGI value at baseline and DKA severity and diabetes microvascular complications.

Variables	DKA Severity			Diabetes Microvascular Complications		
	OR *	95% CI	*p* Value	OR *	95% CI	*p* Value
WBC	1.75	1.10–2.77	0.017	1.13	0.65–192	0.671
Venous blood pH	0.07	0.02–0.21	<0.001	1.52	0.76–3.04	0.233
PvCO_2_	0.56	0.32–0.99	0.049	0.49	0.24–1.03	0.059
Base Excess, mmol/L	0.03	0.01–0.14	<0.001	1.32	0.82–2.16	0.252
Lactate, mmol/L	1.86	1.17–2.96	0.009	0.79	0.43–1.17	0.469
Bicarbonate, mmol/L	0.17	0.07–0.42	<0.001	0.67	0.35–1.26	0.212
Anion gap, mEq/L	1.69	1.01–2.86	0.047	1.52	0.83–2.76	0.169
LGI	1.87	1.13–3.13	0.016	2.16	1.20–3.87	0.010

* OR expressed per 1 SD increase in baseline LGI.

## Data Availability

The datasets used and/or analyzed during the current study are available from the corresponding author upon reasonable request.

## Abbreviations

The following abbreviations are used in this manuscript:

DKA	Diabetic Ketoacidosis
ADA	American Diabetes Association
ICU	intensive care unit
IL	interleukin
SD	Standard Deviation
bpm	beats per minute
SBP	systolic blood pressure
DBP	dystolic blood pressure
BUN	blood urea nitrogen
